# Epidemiology of alcohol dependence in UK primary care: Results from a large observational study using the Clinical Practice Research Datalink

**DOI:** 10.1371/journal.pone.0174818

**Published:** 2017-03-31

**Authors:** Andrew Thompson, Alison K. Wright, Darren M. Ashcroft, Tjeerd P. van Staa, Munir Pirmohamed

**Affiliations:** 1 Wolfson Centre for Personalised Medicine, Institute of Translational Medicine, University of Liverpool, Liverpool, United Kingdom; 2 Centre for Pharmacoepidemiology and Drug Safety, University of Manchester, Manchester Academic Health Sciences Centre (MAHSC), Manchester, United Kingdom; 3 Health eResearch Centre, Farr Institute, University of Manchester, Manchester, United Kingdom; 4 Division of Pharmacoepidemiology & Clinical Pharmacology, Utrecht University, Utrecht, The Netherlands; Harvard Medical School, UNITED STATES

## Abstract

This study aims to investigate the incidence and annual presentation rates of alcohol dependence in general practice in the UK, and examine age-, gender-, socioeconomic-, and region-specific variation. We conducted a retrospective 'open' cohort study using the Clinical Practice Research Datalink (CPRD), an anonymised primary care database. Prior to data extraction, a case definition for alcohol dependence in CPRD was established using 47 Read codes, which included primary alcohol dependence and consequences of alcohol dependence. Directly standardised rates for incidence and annual presentation were calculated for each year between 1990 and 2013. Rates were compared by gender, age, UK home nation, and practice-level Index of Multiple Deprivation. The directly standardised annual incidence rates were 8.3 and 3.7 per 10,000 male and female patients, respectively. The estimated annual rates of presentation per 10,000 were 17.1 for males and 7.6 for females. Female to male rate ratios were: 0.40 (95% CI: 0.39–0.41) for incident cases; and 0.37 (95% CI: 0.36–0.39) for annual presentation. Rates were highest in those aged 35–54 for both measures and across genders, and lowest in those aged over 75 years. With England as the reference nation, Northern Ireland and Scotland had significantly higher rates for both measures. Patients from the most deprived areas had the highest incidence and annual presentation rates. There is unequal distribution of patients with severe alcohol dependence across population subgroups in general practice. Given the health and economic burden associated with dependent drinking, these data will be useful in informing future public health initiatives.

## Introduction

Alcohol dependence manifests from chronic, repeated exposure to ethanol which results in a cluster of behavioural, neurological, and physiological adaptations [1]. The condition is complex with heterogeneous symptoms and various degrees of severity. However, it is well established that consumption of alcohol at the levels synonymous with dependence has substantial negative impact on the individual [2] and society at large [3, 4]. Due to the health, social and wider economic burden associated with alcohol dependence, it is important to have accurate and up to date information relating to disease epidemiology to ensure adequate national and regional resources are allocated for current and future treatment and support.

In the United Kingdom (UK), most of the evidence on alcohol dependence has arisen from large-scale population surveys that utilise screening tools, such as the alcohol use disorders identification test (AUDIT) or severity of alcohol dependence questionnaire (SADQ), and/or quantity frequency methods rather than clinical diagnoses. Such an approach can be time efficient and enable substantial coverage, but can also lead to biases: Selection bias can arise as patients from certain populations are not included (e.g. homeless, university students occupying halls of residents, and military personnel); responder bias from participants knowingly or unknowingly misreporting—for example, it is estimated that self-reported alcohol consumption underrepresents alcohol sales by 40–60% [5, 6]; and, non-response bias [7]. However, the most recent national survey, which was undertaken in England, estimates prevalence of alcohol dependence to be 5.9% [8].

Here we use a longitudinal primary care database, the Clinical Practice Research Datalink (CPRD), to investigate the epidemiology of alcohol dependence in general practice in the UK. Over 98% of the UK population is registered with a general practice. Therefore, General Practitioners (GPs) are likely to be well placed to identify [9] and possibly treat [10] patients with alcohol dependence, but national data in the UK is lacking. A previous study has utilised CPRD to identify the prevalence of alcohol use disorders, including dependence, and reported low identification rates compared with the 2000 Office for National Statistics (ONS) Survey of Psychiatric Morbidity [11]. However, the Cheeta study only reported prevalence in patients who attended their GP during 2003, which means a significant number of patients who were previously or subsequently diagnosed, may have been omitted. Furthermore, we have undertaken a comprehensive process to produce a case definition for alcohol dependence in CPRD. The specific aims of this study were:

1. Present the methods and codes used to develop our case definition of alcohol dependence in CPRD.
2. Explore incidence and annual presentation rates of alcohol dependence between 1990 and 2013 using clinical codes.
3. Define how rates are influenced by gender, age, UK nation, level of deprivation, and calendar year.

## Materials and methods

### Data source

The data was sourced from the Clinical Practice Research Datalink (CPRD), a large database which contains anonymised primary care data from about 8% of the UK population. CPRD has been shown to be broadly representative of the UK population [12] and at the time of data analysis covered 689 contributing general practices within the UK. Data in CPRD is routinely collected and includes patient demographic information, diagnoses, hospital referrals, prescription details, laboratory test results, and lifestyle variables such as smoking status and body mass index (BMI). Quality checks are performed on all data to ensure that they reach the required standards for inclusion. Detailed information on CPRD is available elsewhere [12]. The study was approved by the Independent Scientific Advisory Committee on 03/11/2015 (protocol 14_151A). Patient and practice confidentiality was maintained in accordance with the CPRD policy on personal data.

### Case definition

Diagnostic information is recorded in CPRD by General Practitioners using a hierarchical system of coding known as Read codes [13]. Due to the large number of coding options available for many of the diagnoses, we undertook a systematic, multistep process to produce a case definition for alcohol dependence when using CPRD:

1. Exploration of the Read code database identified an initial list of 289 codes of interest, of which 144 were excluded after review because of lack of relevance or potential to indicate alcohol dependence (e.g., Alcoholic paranoia);
2. Following extraction of the remaining codes in CPRD, we undertook several queries relating to code frequency. We also explored patients that only had a single record of the Read codes under investigation. This exercise resulted in the decision to remove all codes relating to screening tools (e.g. Fast Alcohol Screening Test and AUDIT), as these appeared many times as the only relevant record, and those codes appearing ≤10 occasions in the entire cohort;
3. All codes which describe an alcohol consumption pattern (e.g., Heavy drinker—7-9u/day) rather than an overt diagnosis were excluded because ‘hard’ clinical diagnoses are more readily recorded in CPRD; 4) Our initial code list included a number of codes that referred to some level of treatment for alcohol misuse. These codes vary from brief interventions to admission to a detoxification facility. It was decided that these codes should only be used in conjunction with other hard codes for alcohol dependence in order to examine treatment patterns rather than for identification as a case, unless clinical consensus deemed otherwise; and 5) Two clinical experts in alcohol/addiction were asked to independently review the remaining 84 codes and dichotomise according to whether they believed the codes likely identified a case of alcohol dependence. Where agreement was clear between reviewers, codes were included or excluded (e.g., Problems related to lifestyle alcohol use) accordingly. Discordant codes were discussed between the reviewers and members of the research team, and grouped according to consensus. This process resulted in 47 codes being included in the case definition for “alcohol dependence”. The clinical code list is available from www.clinicalcodes.org [14] and S1 Table.

### Study design and population

We utilised an 'open' cohort study design, such that each patient's time at risk commenced at a different time point, and some exited prior to the end of the study period. The study population consisted of all individuals in CPRD aged 16 years or older between 1 January 1990 and 31 December 2013. This age restriction was set because despite the legal age of alcohol consumption in the UK being 18 years, the National Institute for Health and Care Excellence (NICE) provide treatment guidance for those aged 16 years and older [15]. Patients were entered into the study cohort if they had been registered with a practice for at least 365 days and had a diagnosis of alcohol dependence. Follow-up ended at either the close of the study period (31 December 2013), transfer of the patient out of the practice or patient’s death, whichever came first.

### Data analysis

Incidence rates, “new presentations”, were calculated for each calendar year from 1990 to 2013. To be eligible for inclusion in a given year, patients had to be registered at the start of the year (1 January), be contributing data to CPRD throughout the year, and have no recorded history of alcohol dependence in CPRD prior to start of the year. For history of alcohol dependence, we explored previous Read codes, and the duration of previous time at risk for each patient was dependent on their time registered at a GP practice that is included in CPRD. Exit from the at-risk population was the earliest date of incident diagnosis (numerators) or 31 December of the specified year (denominators). Patients were excluded from the denominator population if the date of death, transfer out of practice or last data collection from the practice was during the given year.

Annual presentation, “one or more presentations of alcohol dependence during the year”, was used to calculate the proportion of patients presenting annually with alcohol dependence, and as an alternative measure of prevalence. To be eligible for inclusion in a given year, patients had to be registered at the start of year (1 January) and be contributing data to CPRD throughout the year. The numerators were estimated as the number included in the denominator with one or more presentation(s) of alcohol dependence during the given year. The same patient-level exclusion criteria was applied.

The analysis for the present investigation focused on identifying alcohol dependence risk between subgroups of the UK population. The initial comparison explored risk in males versus females. We subsequently conducted gender-specific comparisons across age bands, UK Home Nations, and practice-level Index of Multiple Deprivation (IMD) 2010 quintiles (1 = least deprived; 5 = most deprived). IMD general practice-level linkage is available for all practices in CPRD. The general practice postcode is linked via lower layer super output areas or datazone in Scotland, and nation-specific IMD scores extracted. These scores are not directly comparable but act as a proxy with broadly similar measures across seven domains: 1) income, 2) employment, 3) health deprivation and disability, 4) education, skills, and training, 5) barriers to housing and services, 6) crime, and 7) living environment. All subgroup rates were directly standardised for age, nation, and deprivation quintile, and are presented per 10,000 patients. Directly standardised rates were calculated for each measure using the stratum-specific proportions from the relevant population distributions of the total eligible CPRD database population.

Formal analysis was undertaken using Mantel-Haenszel risk ratios, stratified by study year, and presented with 95% confidence intervals. Logistic regression was used to test for temporal linear trends, but this data is not reported due to observing no significant differences for all covariates. We tested the association between incidence and annual presentation against ONS alcohol-related mortality figures for 1994 onwards [16], stratified by gender. The first differences approach was utilised to account for trend before calculating correlation coefficients using linear regression models. Statistical significance was set at *P*<0.05. All data analyses were conducted using Stata version 13 [17]. Strengthening the Reporting of Observational Studies in Epidemiology (STROBE) guidelines were utilised, where applicable (S2 Table)

## Results

### Gender and age

**Fig 1:**
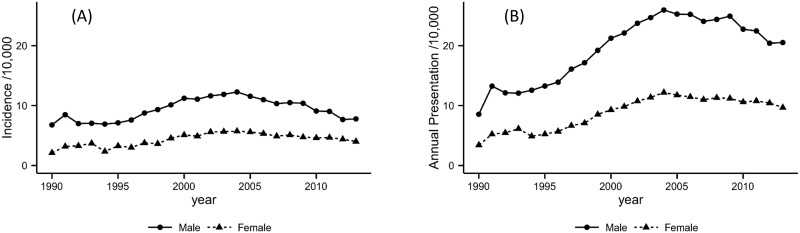
Overall incidence (a) and annual presentation rates (b) of alcohol dependence.

When our case definition was applied to the complete CPRD database, we identified 128,174 cases of alcohol dependence. The directly standardised annual incidence rates were 8.3 and 3.7 per 10,000 male and female patients, respectively. The estimated annual rates of presentation per 10,000 were 17.1 for males and 7.6 for females. When formal analysis was conducted, the following female to male rate ratios were produced: 0.40 (95% CI: 0.39–0.41) for incident cases; and 0.37 (95% CI: 0.36–0.39) for annual presentation. Over time, the rates for both measures were consistently higher in males (Fig 1). There is evidence to suggest that males had higher rates of incident events and annual presentation in each of the subgroups for age, nation and deprivation.

**Fig 2:**
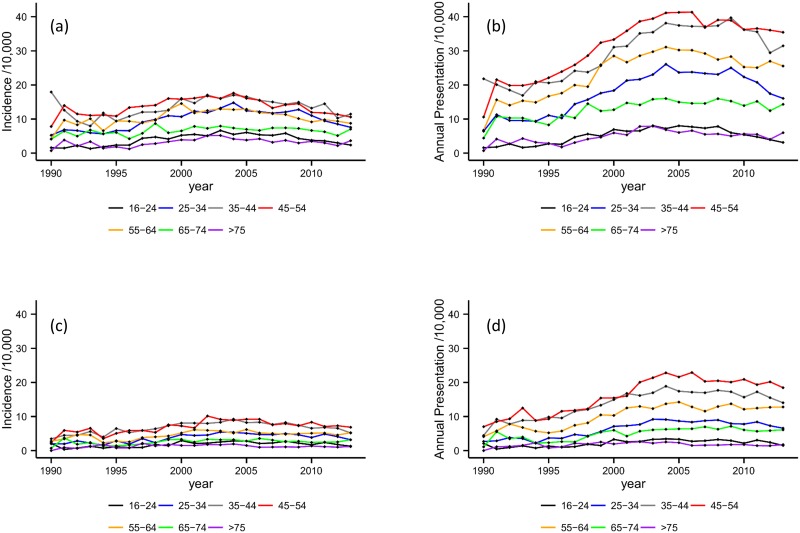
Incidence and annual presentation rates by age band. **2a**. Incidence in male patients by age band. **2b**. Annual presentation rates in male patients. **2c**. Incidence in female patients by age band. **2d**. Annual presentation rates in female patients by age band.

Age-specific rates are presented in Table 1 and illustrated in Fig 2. Generally, rates were lowest in younger and older age with the peak years for both incidence and presentation occurring between 35 and 54 years. However, when compared with the reference group, 16–24 years, the risk ratios were significant for each subgroup. For both genders and measures, the risk ratio was significantly higher for all age bands expect 75+ years where it was significantly lower.

**Table 1:** Risk ratios by gender, age, nation of the UK, and deprivation quintile.

	Male			Female		
	Rate/10k patients *	Risk ratio (95% CI) **		Rate/10k patients *	Risk ratio (95% CI) **	
**Incident events**						
Gender	8.3	-	-	3.7	0.40	(0.39,0.41)
Age:						
16–24	3.5	-	-	1.5	-	-
25–34	8.5	1.45	(1.41,1.50)	3.1	1.29	(1.26,1.33)
35–44	12.1	2.10	(2.05,2.15)	5.8	1.57	(1.52,1.61)
45–54	12.4	2.25	(2.21,2.29)	6.3	1.64	(1.61,1.69)
55–64	9.6	1.67	(1.62,1.71)	4.1	1.31	(1.28,1.35)
65–74	5.9	1.23	(1.18,1.27)	2.3	1.11	(1.07,1.14)
75+	2.7	0.64	(0.58,0.70)	1.1	0.81	(0.79,0.83)
Nation:						
England	7.3	-	-	3.3	-	-
N. Ireland	19.5	2.24	(2.17,2.32)	7.2	2.04	(1.98,2.11)
Scotland	16.4	2.14	(2.10,2.18)	6.3	1.86	(1.76,1.96)
Wales	8.0	1.30	(1.27,1.33)	3.6	1.46	(1.41,1.51)
Deprivation:						
1 (least)	6.0	-	-	2.8	-	-
2	7.9	1.23	(1.20,1.26)	3.3	1.10	(1.06,1.14)
3	7.4	1.17	(1.15,1.21)	3.4	1.16	(1.12,1.21)
4	8.5	1.41	(1.38,1.44)	3.5	1.26	(1.22,1.31)
5 (most)	11.8	2.04	(1.99,2.09)	4.8	1.71	(1.66,1.77)
**Annual presentations**						
Gender	17.1	-	-	7.6	0.37	(0.36,0.39)
Age:						
16–24 (1)	4.4	-	-	1.8	-	-
25–34 (2)	15.3	1.92	(1.88,1.96)	5.2	1.49	(1.46,1.52)
35–44 (3)	26.2	3.13	(3.09,3.17)	11.9	2.50	(2.46,2.54)
45–54 (4)	28.6	3.27	(3.22.3.32)	14.5	2.90	(2.87,2.93)
55–64 (5)	21.4	2.85	(2.80,2.90)	9.1	2.10	(2.07,2.13)
65–74 (6)	11.6	1.64	(1.61,1.68)	4.3	1.46	(1.44,1.48)
75+ (7)	4.0	0.85	(0.82,0.89)	1.4	0.75	(0.73,0.78)
Nation:						
England	14.6	-	-	6.4	-	-
N. Ireland	46.1	2.59	(2.53,2.65)	15.6	2.11	(2.07,2.16)
Scotland	38.5	2.31	(2.27,2.34)	14.1	2.09	(2.01,2.17)
Wales	15.0	1.21	(1.19,1.24)	6.8	1.32	(1.28,1.36)
Deprivation:						
1 (least)	12.8	-	-	5.8	-	-
2	15.8	1.14	(1.12,1.16)	6.8	1.05	(1.02,1.08)
3	14.4	1.13	(1.11,1.15)	6.4	1.04	(1.02,1.08)
4	16.8	1.35	(1.32,1.37)	6.8	1.09	(1.07,1.13)
5 (most)	26.7	2.06	(2.02,2.10)	10.3	1.70	(1.65,1.74)

CI = Confidence interval.

* Rates standardised by age band, geographical region and deprivation quintile.

** Mantel-Haenszel risk ratios, stratified by study year.

### Nation

**Fig 3:**
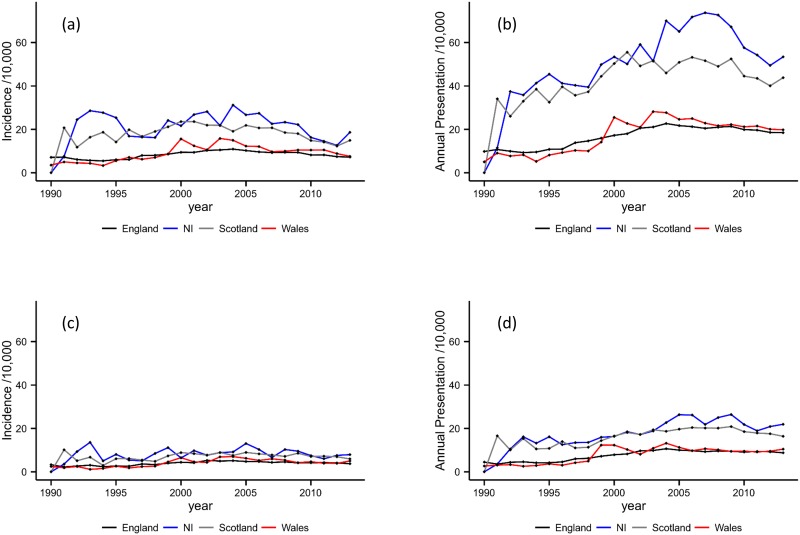
Incidence and annual presentation rates by UK nation. **3a**. Incidence in male patients by nation. **3b**. Annual presentation rates in male patients by nation. **3c**. Incidence in female patients by nation. **3d**. Annual presentation rates in female patients by nation.

Fig 3 presents rates of alcohol dependence stratified by UK nation. Northern Ireland had the highest rates of incidence and annual presentation, followed by Scotland then Wales. Both measures were lowest in England. Generally, the rates in Northern Ireland and Scotland were considerably greater. For example, formal analysis in males produced the following Northern Ireland to England rate ratios: 2.24 (95% CI: 2.17–2.32) for incident cases, and 2.59 (95% CI: 2.53–2.65) for annual presentation; and Scotland to England: 2.14 (95% CI: 2.10–2.18) for incident cases, and 2.31 (95% CI: 2.27–2.34) for annual presentation. Again, there was evidence to suggest a significant variation in trends over time and the absolute changes over time in Northern Ireland and Scotland appear to have greater variation than those in England and Wales.

### Index of multiple deprivation

**Fig 4:**
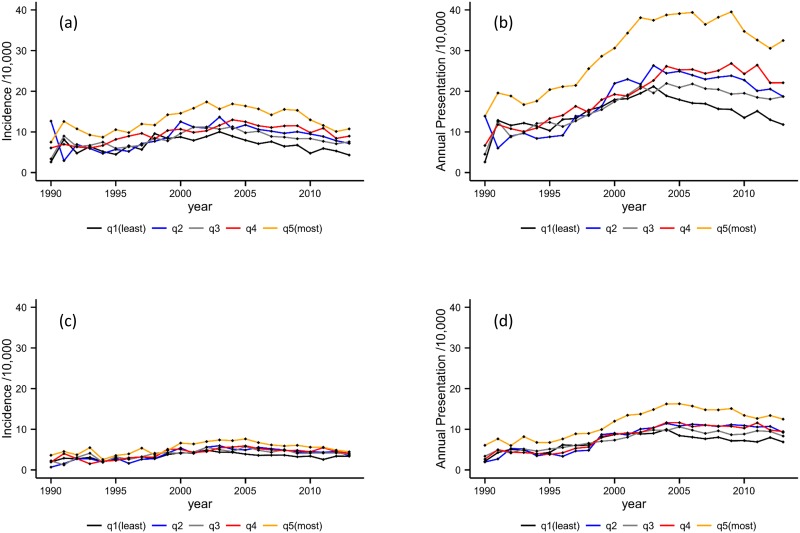
Incidence and annual presentation rates by deprivation quintile. **4a**. Incidence in male patients by deprivation quintile. **4b**. Annual presentation rates in male patients by deprivation quintile. **4c**. Incidence in female patients by deprivation quintile. **4d**. Annual presentation rates in female patients by deprivation quintile.

Fig 4 illustrates rates of alcohol dependence stratified by practice-level deprivation quintile. A positive association between the level of deprivation and incidence and annual presentation rates of alcohol dependence is visible (i.e., as deprivation increases so does the rate of alcohol dependence). Comparing incidence among patients in the most deprived quintile to those in the least deprived quintile, we observed risk ratios of 2.04 (95% CI 1.99–2.09) for males and 1.71 (CI 1.66–1.77) for females. Similarly, the annual presentation risk ratios were 2.06 (95% CI 2.02–2.10) and 1.70 (95% CI 1.65–1.74), respectively. This demonstrates that the magnitude of the social gradient is greater in males than females. The differences for both measures between the mid-range quintiles (i.e. 2, 3 and 4) were less pronounced, and there was a general trend towards those in the second quintile having higher rates than the third quintile across both measures.

### Association with mortality

Incidence and annual presentation rates were significantly correlated with alcohol-related mortality in females (*P* < 0.01) with correlation coefficients of r = 0.59 and r = 0.63, respectively. Annual presentation rates were significantly correlated with alcohol-related mortality in males (*P* = 0.045) with a correlation coefficient of r = 0.47; incidence rates regressed against alcohol-related mortality approached statistical significance (*P* = 0.051) and had a correlation coefficient of r = 0.45.

## Discussion

We found rates of alcohol dependence in UK primary care to be below 1% for both incidence and annual presentation; this is lower than overall estimates using the general population. In 2000 the estimated prevalence of alcohol dependence in Great Britain in the six months prior to data collection was 7.4% overall and 11.9% and 2.9% for men and women, respectively in 2007 [8]; the corresponding figures from a similar survey in England were 5.9%, 8.7% and 3.3% [18]. The majority of cases identified had mild dependence, with only 0.5% of each survey population having moderate or severe dependence. These latter categories are more likely to be representative of our cohort from primary care, because of our case definition, and thus provide a more accurate comparison. Our case definition was developed through collaboration with clinical experts, and is designed to have high specificity for alcohol dependence, meaning our cases are more likely to have more severe forms of the disease. This decision was taken to ensure that our cohort was not diluted by individuals who had non-dependent drinking alcohol use disorders (e.g., hazardous or harmful drinkers). The Alcohol Needs Assessment Research Project also used the data from the 2000 survey but adjusted the criteria for alcohol dependence resulting in a prevalence of 3.6% overall, 6.0% for men and 2.0% for women [19]. The gender-specific effect we observed is consistent to that reported in the population surveys. The gender gap we observed remained relatively stable for both measures, which is contrary to some reports that suggest the gap is narrowing in some developed nations [20].

We found age to be an independent risk factor for alcohol dependence, with similar age group patterns when stratified by gender. However, consensus on which age group is most susceptible has not been reached. Many trials investigating interventions for alcohol dependence report a mean sample age of mid to late 40’s [21]. By contrast, population surveys suggest that younger age groups are at greater risk. This could be due to people not seeking medical help until their alcohol consumption significantly impacts their health [22]. Alternatively, younger people may be more likely to be dependent on alcohol, as indexed by survey criteria, but less likely to perceive the need for treatment [23]. Delays in seeking treatment for alcohol misuse may be associated with cultural norms of heavy drinking [24]. Despite the general inverse relationship between alcohol consumption and age, those that misuse alcohol during late adolescence / early adulthood are more likely to suffer negative consequences later in life [25]. Alcohol-related mortality data generally supports our findings of the greatest impact of alcohol being between the ages of 40–75 years, but as expected, a time lag between measures of alcohol dependence and mortality appears to be evident.

The association between the risk of alcohol misuse/dependence and higher deprivation is well established [26]. Despite those from more deprived backgrounds being more likely to abstain, they are more likely to be negatively affected by alcohol if they do drink [27]. This was evidenced in the recent Local Alcohol Profiles for England dataset where areas with higher proportions of deprivation were more likely to have increased rates of alcohol-related hospital admissions and mortality [28]. Although health is often adversely affected during times of recession, our findings suggest that there has been a steady decline in incidence and annual presentation of alcohol dependence since 2005. The general pattern we observed for these measures are supported by variation in sales data, a proxy for alcohol consumption [29], and decreasing alcohol-related mortality between 2008 and 2013 [16]. A similar pattern of reduced alcohol and nicotine use in heavy users has been reported in previous periods of national financial instability [30]. However, a greater than 3% increase in national unemployment has been associated with a 23% increase in mortality from alcohol abuse [31]. Data from the United States suggest that both abstinence and binge drinking increased during 2008–09 [32]. The long term-effects of the recent recession on alcohol consumption and subsequent morbidity and mortality in the UK will require exploration.

Northern Ireland and Scotland were nations with the highest rates of alcohol dependence. England was the nation with lowest risk for both measures, although empirical evidence suggests that there is regional variation, with North West and North East England having considerably higher rates than the East Midlands, for example [28]. These national level results are consistent with rates of adult smoking (no data available for NI) [33], self-harm [34], and alcohol-related mortality [16]. As each nation is broadly responsible for its own health agenda since relevant devolution in 1998 [35], there are subtle variations in alcohol polices. Scotland, for example, introduced a national alcohol brief interventions programme in 2008 and continues attempts to implement minimum unit pricing, which has been proposed as an effective strategy to reduce consumption in those who purchase low cost per unit alcohol [36], whilst having minimal impact on low/moderate drinkers [37]. Whether this policy is implemented across the UK will likely depend on the outcome of the ongoing legal processes in Scotland [38]. Also, other nations may postpone implementation until evaluations are undertaken on the subsequent impact on consumption and alcohol-related morbidity and mortality if the policy is implemented.

The only other study to use CPRD for a similar purpose reported low identification of alcohol dependence compared with the Psychiatric Morbidity Survey [11]. Although our findings are suggestive that identification is low compared with surveys, we are not able to provide a direct comparison because of a different study time frame and lack of knowledge regarding the Read codes used. The study from Cheeta and Colleagues [11] did report that females who were dependent on alcohol were proportionately more likely to be identified than men, which, if it holds true, is important given that females suffer more negative physical consequences from excessive alcohol consumption [39].

Major strengths of this study include the size of the population from which the sample is taken, the case definition adopted, and the representation from four nations. Our study also has limitations. First, although we view the process adopted to create our case definition for alcohol dependence as a strength, we also recognise that it may have limitations. The definition was designed to have high negative predictive value, and thus will likely capture patients with more severe alcohol dependence and will miss those with milder manifestations of the condition. We also acknowledge that our annual presentation measure will only capture the treatment-seeking population, meaning that only a point prevalence is provided. This may have led to an underestimation of the incidence and annual presentation when alcohol dependence is defined according to NICE criteria [15], which uses formal assessment tools such as AUDIT and SADQ for identification and grading of severity. This is reflected in the estimated prevalence of alcohol dependence in population surveys; however, moderate and severe dependence accounts for a small proportion of the overall cases, thus making us more confident that our case definition is fit for purpose. Second, CPRD is a database that reflects primary care, and any interpretation of the findings needs to consider the data source. Whether the reported rates map directly to the general population is somewhat equivocal given that CPRD represents a cross-section of the population. Furthermore, Theoretical and empirical evidence suggests that many patients who are dependent on alcohol may attend secondary or tertiary services to receive either planned or emergency treatment that is related to their alcohol consumption. Although not unique, this treatment pattern means that patients on these pathways require GPs to follow-up and subsequently record any diagnosis in their primary care records to be eligible for our cohort. Third, data for deprivation status was obtained at practice level rather than for each individual. Although practices are shown to be generally representative of their registered population, having individual deprivation status would have been informative due to the strong association with alcohol dependence. Patient postcode level data is available from CPRD but this only represents practices in England that have consented to linkage, which is a small ecological area of the UK. Finally, we are restricted in our interpretation of incidence rates, as we rely upon the first diagnosis recorded in general practice. There are possibilities that patients may have had alcohol dependence prior to either seeking medical help or registering with the practice. Furthermore, the decision to only include those only registered for with a practice for at least 365 days might have resulted in a form of selection bias.

In summary, our findings provide further evidence for the unequal distribution of alcohol dependence across population subgroups. This adds to the wealth of data collected and disseminated on groups most at risk of alcohol-related health problems, but we have limited knowledge on how best to manage this patient cohort, although public health policies may be most effective if they target these higher risk groups Future research needs to provide insight into how patients engage and move through the current care pathway. This includes evidence on treatment practices and factors associated with negative outcomes (e.g. return to drinking and progression to end-stage disease), and datasets that include understudied populations (e.g. homeless).

## Supporting information

S1 Table Read codes included in the case definition for “alcohol dependence”. (DOCX)

S2 Table STROBE statement checklist. (DOC)
